# Surgical Management of Multijoint Septic Arthritis due to Rat-Bite Fever in a Pediatric Patient: A Case Study

**DOI:** 10.1155/2017/2183941

**Published:** 2017-01-31

**Authors:** Adam M. Wegner, Nicole Look, Brian M. Haus

**Affiliations:** ^1^University of California Davis Medical Center, Sacramento, CA, USA; ^2^University of Colorado School of Medicine, Aurora, CO, USA

## Abstract

In the United States, rat-bite fever is a rare systemic illness principally caused by* Streptobacillus moniliformis*, an organism found in the nasopharyngeal flora of rodents. Infection through direct exposure to rat excreta such as saliva, urine, or feces can lead to fever, rash, and an asymmetric migratory polyarthritis. As rodents are becoming more popular as pets, more pediatric cases are being documented. We report a pediatric case of delayed onset septic arthritis in the left wrist and right knee due to* S. moniliformis* from a rat bite. Previously reported pediatric case studies of suppurative arthritis due to* S. moniliformis* have only involved the hip. This case study demonstrates the importance of a thorough exposure history and consideration of zoonotic infections as a cause of septic arthritis in a pediatric patient that requires antibiotics and surgical intervention.

## 1. Introduction

*Streptobacillus moniliformis*, a Gram-negative bacillus, is the leading cause of rat-bite fever in the United States [1]. The organism colonizes the nasopharyngeal flora of numerous rodents, commonly rats, and can be excreted in the urine [2, 3]. Upon exposure to a rodent bite or scratch, the risk of infection is approximately 10%, with a mortality rate of about 13% if left untreated [1, 3]. The illness is termed “Haverhill fever” when it occurs in an epidemic outbreak caused by exposure to contaminated food or water [4–6].

Rat-bite fever can present acutely or follow a relapsing and remitting course, with most cases spontaneously resolving after 2 weeks [4, 7]. The illness is characterized by a triad of irregularly relapsing fever, delayed onset erythematous maculopapular rash, and asymmetric polyarthritis [2, 4, 5, 8]. Additional manifestations include cough, chills, headache, nausea, vomiting, myalgia, and minimal regional lymphadenopathy [5]. On rare occasions, rat-bite fever may be complicated by a brain abscess, splenic or renal infarction, meningitis, fulminant sepsis, pericardial effusion, endocarditis, myocarditis, pneumonia, destructive joint disease, amnionitis, or death [3, 5]. When patients present with the systemic illness due to a rat bite, the bite site is typically well healed without residual inflammatory reaction [8].

As pet rats and other rodents are becoming more popular, rates of rat-bite fever are rising [6]. Children represent over 50% of the reported cases [6, 9]. Most previous case reports regarding pediatric patients with septic arthritis due to* S. moniliformis *involve a single joint, primarily the hip. We report a case of septic arthritis at the left wrist and right knee due to* S. moniliformis* in a 4-year-old patient.

## 2. Case Presentation

A 4-year-old male presented with a three-day history of fever to 103°F and left wrist pain. His parents reported that he had been guarding his wrist and refused to use it during his daily activities. They also reported a petechial rash on the soles of his feet that had resolved on presentation. On examination, the patient was afebrile and refused to actively move his left forearm and wrist and had pain with wrist flexion/extension and forearm pronation/supination.

**Figure 1:**
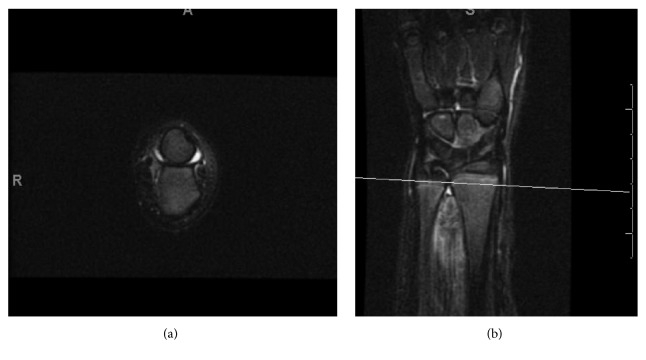
(a) Axial T2 fat suppressed and (b) coronal STIR MRI of the left wrist showing fluid in the distal radioulnar joint (DRUJ).

Full blood examination was nonrevealing. The leukocyte count was 5.1 k/mm^3^ (reference range: 5.5–15.5 k/mm^3^), the C-reactive protein level was 0.5 mg/dL (0–0.8 mg/dL), and the erythrocyte sedimentation rate was 8 mm/HR (0–13 mm/HR). His C-reactive protein level was reportedly elevated at an outside hospital. Wrist arthrocentesis was performed with minimal fluid obtained, with a leukocyte count of 31/mm^3^. Due to continued pain, an MRI of the left wrist was obtained (Figure 1), revealing fluid in the left distal radioulnar joint (DRUJ).

The patient underwent formal irrigation and debridement of the left DRUJ through a dorsal approach. Purulent fluid was obtained and sent for Gram stain and culture. No organisms were found on Gram stain, and the patient was started on empiric vancomycin and ceftriaxone, placed in a short arm splint, and made non-weight-bearing to the left upper extremity for soft tissue rest. Throughout the hospitalization, he remained afebrile. On postop day 2, the pediatric infectious disease team discovered that a pet rat had bitten the patient several weeks earlier to presentation. For this, antibiotics were switched to intravenous penicillin G. An ASO titer was also sent to rule out postinfectious reactive arthritis, which came back negative.

On the fourth day of hospitalization, the patient began refusing to bear weight on his right leg with physical therapy. On exam, he was holding the knee in a flexed position, the knee was warm, and a moderate effusion was present. An arthrocentesis of the knee revealed turbulent fluid and a leukocyte count of 75,250 31/mm^3^. Again, no organisms were seen on Gram stain. The right knee was formally irrigated and debrided with non-weight-bearing restrictions postoperatively for soft tissue rest. Cultures of fluid obtained from the DRUJ also came back that day growing* S. moniliformis*, confirming the rat-bite fever diagnosis. In total, the patient underwent a 7-day course of intravenous penicillin G and a 7-day course of oral penicillin. At his one-week follow-up clinic visit, he was afebrile and with no arthralgias. Sutures were removed and the left arm and right leg were advanced to weight bearing as tolerated with no restrictions after having been non-weight-bearing since operative I&D.

## 3. Discussion

This case report shows that the presentation of septic arthritis from* S. moniliformis *in children can vary. As discussed in previous case reports and reviews, involvement can be monoarticular or polyarticular and may affect either large or small joints [4]. In adults, it has been reported that peripheral joints are more likely to be involved than axial joints with the knees, ankles, elbows, and shoulders as the most commonly affected joints. In contrast, pediatric patients primarily demonstrate infected hip joints [8, 10]. Hambridge and Ogle reported a case of a 16-year-old male with involvement of the knee, shoulder, and ankle and a synovial fluid analysis significant for a leukocyte count of 34,000 [11]. In contrast to our case study, the 16-year-old male did not undergo arthrotomy and debridement of the affected joints.

The pathogenesis of the polyarthritis remains controversial. It has been postulated that two mechanisms may be involved: immunological and inflammatory [1, 4]. Analysis of radiological and clinical data suggests that* S. moniliformis *may tend to damage physes and acrophyses with a predilection for synovial and serosal surfaces [1, 7]. Savage et al. experimentally simulated progressive polyarthritis in mice from* S. moniliformis* that initiated as a fibrinopurulent exudate within the joint and adjacent periosteum. After 4 days, macrophages predominated the infection site, eventually leading to a periarticular abscess and necrosis [12]. Furthermore, the suppurative arthropathy found in review of multiple case studies supports direct inoculation of the joint leading to an inflammatory response [4, 7]. It is noted that the organism may persist in the joint space at 3 months of infection even if the organism has been cleared from the blood, liver, and spleen [1]. Thus, the onset of joint involvement typically occurs around the fourth day of symptoms and may persist for months [2].

*S. moniliformis *is characterized as a highly pleomorphic, filamentous, Gram-negative, nonmotile, and non-acid-fast rod [1]. The most common isolate source in human infections is blood [6]. While it is important to extract joint fluid for analysis, Gram-staining the joint fluid may only contain numerous polymorphonuclear leukocytes without organisms. Despite optimal conditions, growth may require up to five days [3]. Growth upon subcultures can reveal occasional clumped highly pleomorphic Gram-negative filaments with numerous bulbous swellings that could be mistaken for proteinacious debris [2]. The most useful confirmatory test is 16S RNA sequencing and DNA mapping [3].

The recommended treatment for streptobacillary rat-bite fever is intravenous penicillin G for five to seven days, followed by oral penicillin or ampicillin for seven days [8]. Patients with penicillin allergies or those infected with the L-form of* S. moniliformis* have been successfully treated with either oral tetracycline or intramuscular streptomycin [8]. Additional treatment involves arthroscopy, arthrotomy, or joint lavage to control localized disease of the joint for all patients who present with septic arthritis of large joints. In children, it is recommended that all joints, large or small, undergo surgical intervention to visualize the joint structure, evaluate the degree of destruction, and formally irrigate and debride the infected joint [8]. Although there is a possible concern of effect on future growth, no studies have demonstrated such outcomes.

Infection caused by* S. moniliformis* after exposure to rats or other rodents can present as an asymmetric polyarthritis that more commonly involves the peripheral joints. In children, the hip is most commonly involved. We recommend a thorough exposure history in all patients that present with unusual or asymmetric polyarthritis. Although diagnosis of septic arthritis typically involves joint aspiration, it can be difficult to isolate* S. moniliformis*. Thus, it is essential to recognize unique qualities of bacterium with repeated examination of the bacterial culture. Treatment consists of a course of intravenous and oral penicillin. For those with penicillin allergies, streptomycin and tetracycline serve as alternatives. In the setting of a pediatric patient, surgical intervention is often necessary leading to an overall good outcome.
